# Advances in Animal Cognition

**DOI:** 10.3390/bs6040027

**Published:** 2016-11-30

**Authors:** Jennifer Vonk

**Affiliations:** Department of Psychology, Oakland University, 654 Pioneer Drive, Rochester, MI 48309, USA; vonk@oakland.edu

**Keywords:** advances, animal, cognition, diversity, species, comparative psychology

## Abstract

This editorial endorses a diverse approach to the study of animal cognition and emphasizes the theoretical and applied gains that can be made by embracing this approach. This diversity emerges from cross-talk among scientists trained in a variety of backgrounds and theoretical approaches, who study a variety of topics with a range of species. By shifting from an anthropocentric focus on humans and our closest living relatives, and the historic reliance on the lab rat or pigeon, modern students of animal cognition have uncovered many fascinating facets of cognition in species ranging from insects to carnivores. Diversity in both topic and species of study will allow researchers to better understand the complex evolutionary forces giving rise to widely shared and unique cognitive processes. Furthermore, this increased understanding will translate into more effective strategies for managing wild and captive populations of nonhuman species.

## 1. Introduction

The study of animal cognition has witnessed enormous growth in the last decade, both with regard to the subject matter and the species investigated. This increased breadth comes about, in part, because of external pressures (e.g., lack of funding, stricter control over captive animal care) and theoretical challenges (e.g., increasing interest in evolutionary forces selecting for cognitive processes). These pressures and challenges have motivated scientists to reach beyond the narrow confines of the lab, and to seek collaborations with sanctuaries, zoos, farms, and pet owners in order to investigate cognition in a wider variety of species than ever before. Furthermore, both funding sources and the climate of the field have encouraged investigators to establish interdisciplinary collaborations [1]. Thus, biologists, anthropologists, zoologists, behavioral ecologists, and comparative psychologists have converged upon the study of animal cognition in order to better understand the mental lives of animals. A multi-disciplinary approach to the topic all but guarantees innovative and impactful approaches to a field that is still relatively young. Knowledge gained from these new methods and approaches can then be used to inform conservation practices for wild animals, and welfare practices for captive species. This Special Issue presents a series of papers that embody the innovation and breadth embraced by scientists currently investigating animal cognition. Papers are presented on species ranging from bumblebees [2], to North Island robins [3], Jamaican fruit bats [4], domestic horses [5], sun bears [6], and non-human primates [7,8,9,10,11]. The research reported in this issue was conducted in a range of wild and captive environments, including laboratories, zoos, and conservation organizations. The goal of the Special Issue is to emphasize the diversity of approaches in the current study of animal cognition and to demonstrate the progress that can be made by embracing this diversity.

## 2. Topics

Comparative cognition, as a discipline, was founded upon the study of basic processes, including perception, categorization, memory, and learning. Smith and colleagues [7] provide an insightful and up-to-date review of the research on categorization in non-humans. They review the primary theories and the ensuing debates and highlight the essential role that animal studies have played in clarifying categorization processes, not just in nonhumans, but in humans as well. This paper exemplifies the goals of the Special Issue in a variety of ways. First, the authors show that a focus on a singular theoretical approach is unlikely to do justice to the topic at hand. Elements of various theories are likely to be important in facilitating categorization across species. Second, they illuminate both continuities and discontinuities between humans and other species, realizing the critical insight that evolution embraces both similarities and differences. Thus, cognitive processes should not be approached from an all-or-none perspective that necessitates the finding of either sameness or difference between species without regard for the notion of pre-cursors or a more modular/mosaic view of cognition. Lastly, they encourage researchers to approach the study of cognition with an appreciation for a fitness perspective. In recent years, researchers have enthusiastically sought evidence for various indices of ‘advanced’ cognition in species with little regard to the potential adaptive function of these capacities in an organism’s evolutionary history [1]. Smith and colleagues [7] remind readers to place their studies within an evolutionary context. Historically, researchers approached the study of cognition tentatively, with the ghost of Behaviorism looming large. Even today, researchers interested in cognitive mechanisms must defend interpretations of their data against the “associative learning” model, as if the formation and generalization of associations between stimuli or between behaviors and outcomes occur in the absence of cognition [12]. Perspectives such as that of Smith and colleagues provide much needed acknowledgement that many processes are not dichotomous, and that associative processes are not the enemy of cognition [13].

Movement away from dichotomous theorizing has led to a shift to more modular accounts of cognition. For example, Subiaul [8] has developed a model of imitation that consists of multiple forms of imitation, e.g., imitation for familiar and unfamiliar actions, and imitation of opaque or intransitive gestures. Subiaul’s model is consistent with the idea that individuals, or species, may have the ability for certain kinds of imitation, but not others. Acknowledging that cognitive abilities may be parsed into separate modules allows for a better basis of comparison between species. Subiaul [8] indicates that human children alone may be capable of imitating novel transitive actions and intransitive actions, while other apes may share the capacity to imitate familiar transitive actions. Rejection of the all-or-none approach to studying species’ differences in cognition represents an advance in understanding which mechanisms may be shared widely in the animal kingdom, and which might be more specialized. Understanding which cognitive facets are unique to particular species will help researchers identify the environmental and social conditions necessary for their emergence in evolutionary history.

Consistent with Subiual’s [8] conclusion, researchers have advanced the idea that humans alone may be capable of representing concepts for constructs that are abstract and unobservable [14]. Reasoning about causal forces may be considered one class of constructs about unobservables. Previously, Vonk and Subiaul [15] demonstrated that even chimpanzees may not reason about causality, even when indicators of capability, for example, are directly observable, such as in the case where human agents’ capability to perform a task depends upon the availability of particular limbs. Garland and Low [3] replicate this study for the first time in North Island robins. Not only is the question of capability an unexplored construct, but few studies have attempted to answer such questions in natural settings. Furthermore, North Island robins have not been extensively studied for their cognitive capacities. Thus, Garland and Low’s contribution represents a significant advance both in terms of delineating the abilities of an understudied species and demonstrating innovative methodology for adapting a lab study to test a wild species in a paradigm that is ecologically relevant. They tested robins’ capacity to reason about human competitors in a natural foraging setting.

In addition to theoretical advances gained from the increasing breadth of topics studied under the broad umbrella of comparative cognition, a better understanding of animals’ cognitive capacities allows for enrichment and welfare programs to be tailored toward assessments of cognitive and emotional well-being, rather than focusing singly on physiological health. Bethell and colleagues’ [11] paper presents an advance in methodology to study cognitive biases in non-humans. Cognitive biases refer to judgement frames whereby animals may be described as optimistic or pessimistic, thus also reflecting an indicator of emotional states. Cognitive bias tests have become popular methods to assess an animal’s emotional well-being, but typical methods require substantial training, and results are often somewhat ambiguous due to complications of interpretation. Bethell et al. [11] present a novel method that requires very little training and does not rely on accuracy of responding to indicate an animal’s well-being. This new method may prove to be very influential in the field of animal welfare, and in fact we have adopted similar methodology to study the welfare of captive gorillas and black bears based on Bethell et al.’s groundbreaking procedure. Perdue [6] also focuses on using cognitive tasks to assess the well-being of captive animals. In her study, sun bears demonstrated a strong interest in cognitive testing as a form of enrichment. This finding is important given the relatively reduced attention given to intellectually stimulating enrichment for carnivores compared to non-human primates in zoological settings. Bears, in general, have been relatively understudied compared to other large-brained mammals with regard to their cognitive abilities, and, within the bear family, very little is known about sun bear preferences or capacities.

Although some of the papers presented in this Special Issue focus on novel questions, others focus on the resolution of current controversies. Parrish and co-authors, [9], for example, fail to show support for the popular glucose hypothesis of self-control, demonstrating the importance of replication efforts. These authors propose that future work investigating the link between self-control and physiological correlates among species varying in phylogenetic distance will be fundamentally important to elucidate the mechanisms underlying self-control. Self-control has recently come to the fore in comparative cognition, as an important aspect underlying intelligence and behavioral flexibility [16], and systematic studies of this capacity in a broad range of species is welcomed.

Linked to the idea of self-control, attentional processes are vitally important for most cognitive functions. Understanding the relationship between attention, inhibitory control, working memory, and general intelligence, will inform research aiming to rank species according to cognitive sophistication and flexibility. Bramlett-Parker and Washburn [10] tested rhesus macaques in a series of cognitive tasks in order to test the idea that attention and other capacities could be enhanced through extended training. Although some improvements with practice were revealed, monkeys did not generalize these improvements to a novel Attention Network Test (ANT), which did not provide strong endorsement of the idea that attentional processes could be modified with experience and training. However, such approaches have both applied benefits by demonstrating the plasticity of cognitive capacities, and theoretical benefits through allowing a better understanding of the relationship between stimulus and cognitive control.

## 3. Species

Like the study of self-control, studies of quantity estimation reveal some aspect of this capacity in a wide range of species. However, quantity abilities had not been extensively studied in equines, or hoofstock of any kind. Petrazzini [5] developed a novel method to investigate quantity judgements in horses. This method can be adapted to study a variety of cognitive capacities, which is an important development considering that little is known about cognition in horses, despite the fact that they are readily available and have been bred to work closely with humans. A focus on domestic species is warranted both because they are more readily available than many exotic species, and because the study of their cognitive capacities will help uncover the effects of domestication, selective breeding, and rearing environment on the sculpting of cognition.

In addition to focusing on species more closely related to humans (e.g., other primates) and social-living mammals (canines and cetaceans) or birds (corvids), researchers should extend their investigations of cognitive capacities to more distantly related species that thrive in unique environments. Honeybees have been shown to demonstrate various aspects of advanced cognition, such as sameness/difference judgements [17] although they are members of the insect class and, thus, far removed from humans. In addition, bees exhibit eusociality, a unique social structure that might give rise to a host of seemingly sophisticated behaviors through chemical manipulations. Here, Thompson and Plowright [2] extend studies of picture/object recognition in bees to the lesser studied bumblebees. This type of study is critically important in determining what animals understand about visual stimuli presented to them in attempts to capture elements of their cognitive processes. Few species have unequivocally demonstrated correspondence between pictures and their real-life referents (see [18,19]), and Thompson and Plowright’s work [2] represents an important and clever methodology for assessing such a capacity in an insect.

Another large but neglected group of animals, at least with regard to studies of their cognitive capacities, are the chiropterans. Hoffmaster and Vonk [4] explore the topic of prosocial preferences in Jamaican fruit bats—a good candidate species for prosocial behavior, given the large number of reports of cooperative behaviors in bats in the wild, and demonstrations of purportedly altruistic behavior of food-sharing in the vampire bat [20]. Although, these authors failed to find evidence for prosocial behavior in this species using the escape paradigm, their study represents a recent shift away from food-sharing paradigms to a method that can be implemented without extensive training, and which might take better advantage of the natural tendencies of social animals to group together. Such methods will be potentially useful in studying other less food-motivated species. 

## 4. Testing Environments

The bats studied by Hoffmaster and Vonk [4] were housed at the Organization for Bat Conservation’s Batzone. Working with such organizations and sanctuaries not only allows researchers to collect valuable data on rare and understudied species, but also provides the opportunity to collaborate with such organizations to educate the public about unappreciated species that are vital to our ecology (see also [21]). Bees, like bats, are vital to the ecosystem and provide many benefits to human populations. Thus, a better understanding of their cognition and behavior can help guide our conservation efforts and motivations. Of course, studying animals in their natural environments, as was done by Garland and Low [3], will perhaps be the most informative, both with regard to understanding the animals’ cognitive capacities, but also the conditions under which such capacities arose. 

It is also critical that researchers collaborate with zoos, which are also involved in conservation and welfare efforts. Perdue’s study [6] represents one example of an effective working collaboration between a zoo and an academic investigator. Subiaul’s work [8] with human children and great apes also takes place in a zoological setting, which allows for both the recruitment of participants and the immediate dissemination of the results of these studies. Researchers will have to become more resourceful in obtaining subjects to participate in their research protocols. There is still much work to be done testing domestic species, which can be accessed more readily. The horse tested by Petrazzini [5] for example, resided in a private riding stable.

Other work takes place in the more traditional comparative psychologists’ laboratories or research centers [2,7,8,9,10,11]. Although the housing of lab animals has become increasingly controversial, and funding for such work has become decreasingly available, these researchers have made many valuable contributions over the years. The current work is just a tiny sampling of these contributions, and such advances would not be possible without such institutions and researchers. It is hoped that the current Special Issue will bring attention to the importance of such diverse approaches and champion the need for their continuation.

## 5. Conclusions

In sum, there is no single best approach to the study of animal cognition. Advances will be made when researchers come together from a variety of backgrounds and theoretical approaches, and when researchers are willing to forge their paths in a variety of available settings, working with sometimes less popular and less well-known species to paint a detailed portrait of the diversity of mental lives on this planet.
